# Paul Drude's Prediction of Nonreciprocal Mutual Inductance for Tesla Transformers

**DOI:** 10.1371/journal.pone.0115397

**Published:** 2014-12-26

**Authors:** Bart McGuyer

**Affiliations:** Department of Physics, Columbia University, 538 West 120th Street, New York, New York, 10027-5255, United States of America; Institute for Materials Science, Germany

## Abstract

Inductors, transmission lines, and Tesla transformers have been modeled with lumped-element equivalent circuits for over a century. In a well-known paper from 1904, Paul Drude predicts that the mutual inductance for an unloaded Tesla transformer should be nonreciprocal. This historical curiosity is mostly forgotten today, perhaps because it appears incorrect. However, Drude's prediction is shown to be correct for the conditions treated, demonstrating the importance of constraints in deriving equivalent circuits for distributed systems. The predicted nonreciprocity is not fundamental, but instead is an artifact of the misrepresentation of energy by an equivalent circuit. The application to modern equivalent circuits is discussed.

## Introduction

The German physicist Paul Drude (1863–1906) contributed significantly to many fields of science during the late 19th and early 20th centuries [1]. In particular, he remains well known for pioneering work in optics and solid-state physics. Less familiar is that late in life Drude published a series of articles [2]–[5] on the physics of Tesla transformers (or Tesla coils), which at the time were important for early radio communication [6], [7]. While these articles are mainly of historical interest today, the article from 1904 is still cited as a primary reference for the conventional equivalent circuit of a Tesla transformer (e.g., [8]–[10]).

Such equivalent circuits (or lumped-element models) are ubiquitous in the study of physical systems, from acoustic resonators [11] to coupled qubits [12]. Importantly, these circuits are widely used to model not only lumped systems that are small compared to the wavelengths of interest, but also distributed systems like Tesla transformers that may not be. This has long been a standard practice in radio and microwave engineering, especially with resonant transmission lines, microwave networks, and inductors [7], [13]–[15]. Most systems modeled by circuits satisfy some form of reciprocity, or broadly, symmetry under the exchange of source and response [16]. For these reciprocal systems, a common assumption today is that their equivalent circuits must also be reciprocal.

However, there is a startling prediction in Drude's 1904 article [4]: *Drude predicts that the mutual inductance for a Tesla transformer should be nonreciprocal* (i.e., 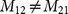). Though nearly forgotten, this prediction seems to have been well known in the early 20th century [17]. Today, it has every appearance of being a mistake. After all, there are no clear sources of nonreciprocity in a Tesla transformer, such as magnetic materials, so how could this prediction possibly be correct? Despite its appearance, we will see that Drude's prediction is indeed true, although for an unexpected reason.

This Article explains the physics behind Drude's overlooked prediction. To proceed, we will not focus on Drude's original derivation of an equivalent circuit for a Tesla transformer. This is because the original unfortunately contains errors and a distracting treatment of inductance. It also neglects to explain the phenomenon behind the prediction. For the interested reader, an English translation and discussion of the original derivation in German has been provided in Ref. 18. Instead, this Article presents a modern treatment of the phenomenon behind Drude's prediction. We will see how reciprocal systems, paradoxically, may have nonreciprocal equivalent circuits in rare applications. Besides historical interest, this phenomenon highlights the boundary between lumped and distributed systems and, in particular, the potential for confusion when modeling the latter with the former.

## Drude's Prediction

**Figure 1:**
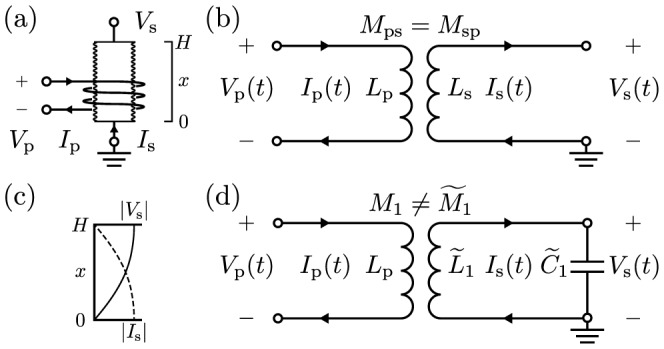
Specific example to illustrate Drude's prediction. (a) Physical setup of primary and secondary inductors with dc self-inductances 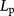 and 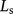. (b) Low-frequency equivalent circuit with reciprocal mutual inductance. (c) Voltage and current spatial profiles for the first self-resonance of the secondary solenoid. (d) Equivalent circuit for frequencies near this resonance with nonreciprocal mutual inductance. This circuit is a simplification of a more complete circuit derived from a distributed-element model treating the solenoid as a transmission line, which is shown in a later figure. All parameters are defined in the text.

To illustrate Drude's prediction, consider the following specific example of an air-core transformer sketched in Fig. 1(a), which could be part of a Tesla transformer. A standard equivalent circuit is sketched in Fig. 1(b) that is valid for direct current (dc) and low-frequency alternating current (ac), assuming the transformer is much smaller than the shortest ac wavelength. For an ideal lumped transformer there are various ways to show that the primary and secondary inductors share the same mutual inductance, 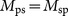, such as reciprocity [19], symmetry [20], and conservation of energy [21]–[23]. In particular, the latter requires this equality because otherwise energy would be lost or gained during transfer between the inductors.

However, what about at higher frequencies? Now let the secondary be a single-layer solenoid, just as in a Tesla transformer. While real solenoids are quite complex [24], they often act very nearly as transmission lines [14], [15], [25]. Following Drude [4], let us then model the secondary as a distributed transmission line. As arranged the solenoid is a quarter-wave resonator. For frequencies near the fundamental self-resonance it will have the current and voltage spatial profiles sketched in Fig. 1(c). While these profiles suggest otherwise, the solenoid in a Tesla transformer is typically much smaller in size than the corresponding free-space wavelength of the fundamental self-resonance. This is because these solenoids are slow-wave structures [15], and near this resonance it is the coiled winding length, which is often enhanced by a large number of turns (e.g., 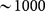), that typically becomes comparable to a quarter wavelength. Nevertheless, the standard "lumped'' circuit in Fig. 1(b) predicts no resonances, and is no longer valid at frequencies near or above the fundamental self-resonance of the solenoid.

We may still derive a lumped-element model (or equivalent circuit) for the transformer, however, by starting with a distributed-element model for the solenoid, just as for a resonant transmission line. Doing this, we will find that for frequencies near the fundamental self-resonance, we may model the voltages and currents in Fig. 1(a) with the equivalent circuit sketched in Fig. 1(d). As derived below, the mutual inductances in this circuit are no longer equal, but satisfy
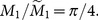
(1)


Surprisingly, conservation of energy requires this result. While Drude's original derivation is incomplete, it may be corrected to give the above result as shown in Ref. 18.

This phenomenon predicted by Drude is an artifact of modeling transmission lines with lumped equivalent circuits. To explain it, we will treat the general case of a uniform transmission line coupled to an external system. We will derive an exact equivalent circuit for the specific example described above, and obtain the simplified circuit in Fig. 1(d) by keeping only the part most important near the fundamental self-resonance, following Drude [4]. The specific result (1) then comes not from any fundamental nonreciprocity, but instead from the subtle choice to model the same voltage and current as in Fig. 1(a), namely the voltage drop across and current into a resonant inductor. It is one example of an artificial nonreciprocity originating from the misrepresentation of energy, or equivalently, from the "lumped'' circuit parameters retaining a distributed character. Finally, we will extend this phenomenon to other equivalent circuits for lines, further examine its application to solenoids and Tesla transformers, and conclude with a discussion.

## Equivalent Circuits for Transmission Lines

**Figure 2:**
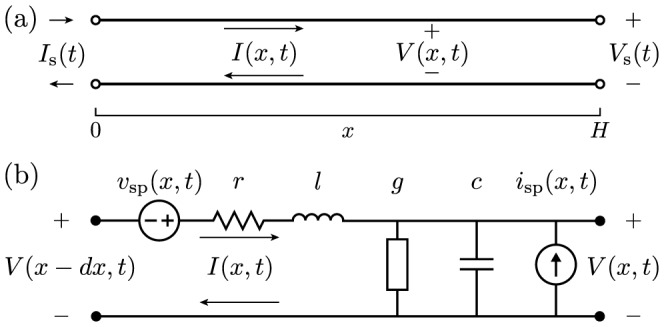
Uniform transmission line described by the Telegrapher's equations (2). (a) Voltage and current conventions and relation to Fig. 1. (b) Distributed-element model equivalent to (2). The equivalent circuits in all other figures are derived from this model.

Consider the transmission line sketched in Fig. 2(a), and described by the four parameters of series resistance 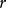, series inductance 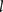, shunt conductance 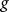, and shunt capacitance 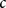, each with units distributed per length. The voltage 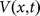 and current 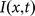 at any position 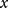 along the line then obey the Telegrapher's equations,
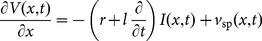
(2a)

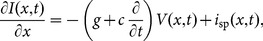
(2b)which correspond to the distributed-element model sketched in Fig. 2(b). The additional terms 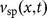 and 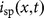 are distributed sources that model coupling with external systems, such as the primary inductor in Fig. 1(a). By convention, positive 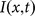 flows towards increasing 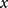 in the solenoid. Before we continue, note that the distributed-element model sketched in Fig. 2(b) is itself a form of equivalent circuit for a line, and that today, unlike with Paul Drude in 1904, there are many numerical methods [26], [27] available to directly use such a model for a line or more complicated systems.

To generate a lumped-element equivalent circuit, we first expand the voltage and current along the line with spatial Fourier series. For the geometry of Fig. 1(a), a convenient choice is the pair of quarter-wave Fourier series
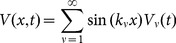
(3a)

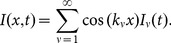
(3b)


For a line of length 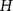, this series is complete in the interior 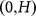 of the line, and the wavenumbers 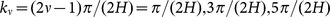, etc.

Next, we introduce a set of lumped circuit parameters 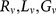, and 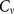 for each spatial mode 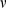 from the corresponding distributed parameters 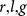, and 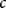, by using the series and shunt scaling lengths
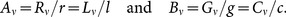
(4)


Any equivalent circuit must preserve the natural resonant frequencies 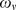  =  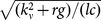  =  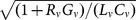 of its modes 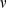, so these scaling lengths must satisfy
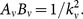
(5)


The lumped parameters 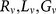, and 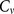 for the mode 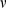 are then determined if we specify the ratio
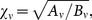
(6)which controls how the circuit represents impedance. From this ratio, 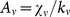 and 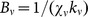. For a given line, there is no unique choice of 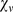 or the resulting parameters 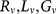, and 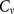. Without loss of generality, however, we can choose the ratio
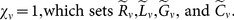
(7)


Here and subsequently, a tilde marks this choice. How to transform to the case of 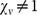 is described below.

Finally, using Eqs. (3–7), the Telegrapher's equations (2) separate to a system of equations for the Fourier amplitudes 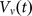 and 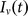 of each spatial mode 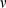,
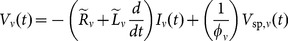
(8a)

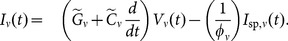
(8b)


Here, the lumped sources that represent coupling with an external system are
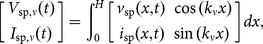
(9)which we will see below is the natural choice from conservation of energy. The angle 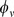 is half the electrical length 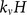 of the line for the spatial mode 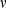,
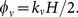
(10)


For the expansion (3), 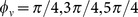, etc.

**Figure 3:**
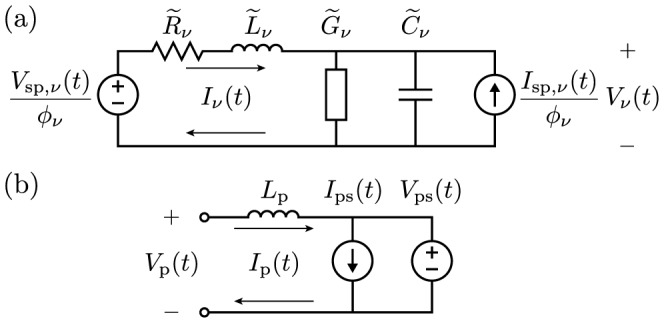
Equivalent circuits for the line and external system. (a) Lumped-element model equivalent to (8) for the 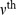 spatial mode. (b) Lumped-element model for a two-terminal port coupled inductively or capacitively with the line. For direct coupling, 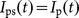 and 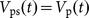. The inductance 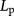 is present only for the specific example. When the line and external system are coupled, the circuits in (a) and (b) combine according to the relationship between the lumped sources.

Together, the set of circuits defined by the system (8), which are sketched in Fig. 3(a), comprise an exact equivalent circuit for the line. Fourier series different than (3) produce similar results, though the nonresonant (dc) terms in some are special cases with 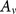 or 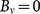. Importantly, note that these separate circuits may stitch together to form one combined circuit depending on the relationship of the sources 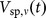 and 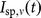 between the modes 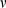.

### Misrepresentation of energy

Before treating coupling in detail, we can explain the nonreciprocity (1) as follows. First, note that the energy stored by the mode 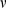 along the line is
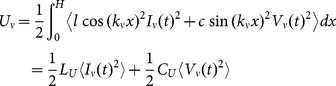
(11)for real-valued 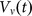 and 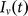. Here, the brackets denote a time average, and the effective parameters are given by 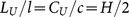. In contrast, the energy modeled by the equivalent circuit (8) is not that of (11), but instead
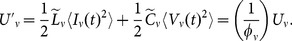
(12)


**Figure 4:**
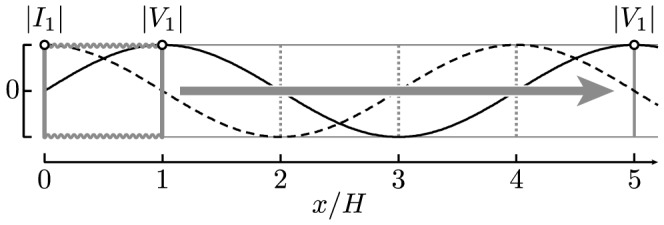
Lengthening argument for the misrepresentation of energy. For 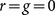 and no coupling, note that two constraints determine 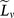 and 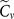: (i) the resonant frequency 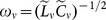, and (ii) the impedance 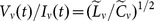. As sketched for 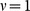, lengthening by one or more wavelengths does not change (i) or (ii), thus neither 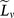 or 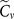. For fixed 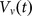 and 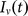, the energy (12) modeled by the circuit in Fig. 2(a) also does not change. However, the stored energy (11) must increase, so this circuit misrepresents energy.

Therefore, the equivalent circuit (8) misrepresents the energy stored (and power dissipated) along the line by a factor of 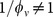. That is, the equivalent circuit (8) models the energy stored per 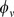 radians along the line. An additional lengthening argument for this misrepresentation is sketched in Fig. 4.

This is the origin of the nonreciprocity (1). Since the equivalent circuit for the line misrepresents energy, its representation of coupling with an external system, such as the primary inductor in Fig. 1, must convert any transferred energy (or power) to this incorrect representation. Assuming the equivalent circuit for the external system represents energy correctly, this conversion requires a directional amplification (or gain) to represent the coupling, which is accomplished by the two factors of 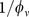 in (8). Amplifiers are nonreciprocal circuit elements, so this representation is nonreciprocal [16].

### Distributed character of circuit parameters

Intuitively, this phenomenon results from the "lumped'' parameters in the circuit (8) still retaining a distributed character: note that 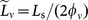 is an inductance per radian just as 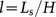 is an inductance per length, where 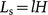 is a dc self-inductance. Thus, for a fixed wavenumber 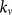, the parameters 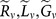, and 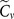 are properties of the line and independent of its length 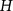, just like 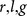, and 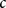. However, for fixed amplitudes 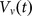 and 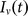, lengthening the line increases its stored energy (11), as sketched in Fig. 4. Therefore, to conserve energy, the equivalent circuit for the mode 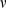 must have a coupling parameter that scales with the length of the line. For the specific example, this is the mutual inductance 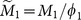 in (1).

### Transformation to other equivalent circuits

So far, we have focused on one particular equivalent circuit. This phenomenon, however, is modified by the choice of circuit, which is not unique. The circuit (8) is constrained to model 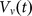 and 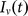, which is appropriate for the specific example because 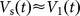 and 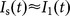 for frequencies near the fundamental 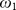. To relate this phenomenon to other equivalent circuits, consider substitutions of the form
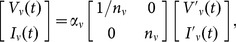
(13)which lead to circuits modeling the variables 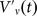 and 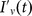. Here, the matrix represents an ideal transformer with turns ratio 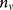. Using (13) with (8) shows that this transformer replaces the parameters 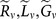, and 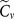 with those set by 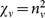. Noting this and using (13) with (12) shows that only substitutions (13) with 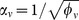 may represent energy correctly. Consequently, all others lead to circuits that have some form of the artificial nonreciprocity described above.

That is, equivalent circuits formed by constraints other than to model energy may exhibit some form of the phenomenon outlined above. Conversely, a reciprocal circuit that models energy may be incompatible with other desirable constraints. As (13) shows, this is the case with the specific example, because modeling energy is incompatible with modeling both 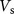 and 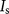 together near resonance. We will treat a more common example with solenoids after finishing the specific example below.

## Coupling with External Systems

Let us now return to model coupling. Consider an external system that is equivalent to a two-terminal port with well-defined voltage 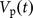 and current 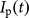, such as that sketched in Fig. 3(b), which could be part of a lumped circuit, for example, or a point on another line. To model coupling with this system, we must treat both the forward and reverse directions, or to and from the line, respectively. To model many common types of coupling simultaneously, let the distributed operators 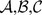, and 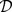 specify the forward coupling as
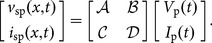
(14)


For the inductive coupling of the specific example,
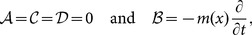
(15)where the function 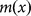 describes the coupling between the inductors, such that 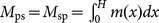. Likewise, the single operator 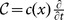 describes capacitive coupling. Direct (or wired) coupling at the bottom 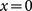 is described by the pair 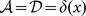, where 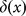 is a Dirac 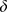 function, and at the top 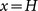 by 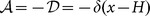. A direct tap at an interior point may be treated by splitting the line into two separate lines with direct couplings at the shared endpoint. Note that direct couplings may modify the boundary conditions modeled by the Fourier series (3).

We may determine the reverse coupling in terms of the coupling operators in (14) as follows. Note that the lumped sources for the port in Fig. 3(b) are sums over contributions from the entire line,
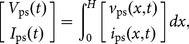
(16)where the distributed sources 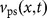 and 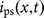 are distinct from 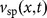 and 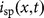 in (2). Assuming the coupling is lossless, passive, and quasistatic, the forward and reverse powers transferred should balance at all 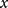,
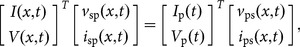
(17)where 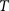 denotes transposition. Using (14), and noting that the coupling operators described above are symmetric for harmonic signals, this gives the reverse coupling
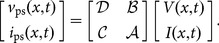
(18)


The off-diagonal operators, which act as a mutual impedance and admittance, are the same as those of (14), as expected from reciprocity. Additionally, 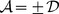 for the couplings described above, so the relations (14) and (18) are equivalent under the exchange of source and response, up to a diagonal sign.

Using (14), the forward lumped sources (9) for each mode 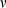 in the expansion (3) are
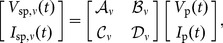
(19)where the coupling operators for the mode 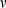 are
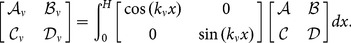
(20)


Likewise, we may write the reverse coupling (16) as a sum over lumped sources from each mode 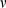,
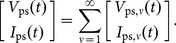
(21)


Using (18) and (20), these lumped sources are
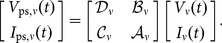
(22)


Following (17), we may use (19) to verify that both sets of lumped sources (9) and (22) conserve power locally,
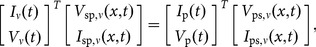
(23)which justifies an earlier assertion for the form of (9). The discussion about reciprocity following (18) also applies here. Note that 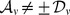 unless both are zero for the couplings considered above.

Together, Eqs. (19–22) specify how the equivalent circuits (8) sketched in Fig. 3(a) couple to the external port, such as sketched in Fig. 3(b). In all cases, the two factors of 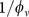 in (8) may be modeled by a directional amplifier (or a nonreciprocal ideal transformer). For inductive and capacitive couplings, this gain can combine with other parameters to simplify the circuit, leading to nonreciprocal mutual inductances or capacitances. For other equivalent circuits, the coupling is given by using (13) with (8) and (19–22), and often involves an ideal transformer.

### Coupling for the specific example

For the specific example, the set of coupling operators (15) leads to a single nonzero mode operator (20),
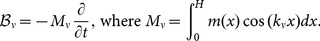
(24)


From Fig. 3(b) and (21–22), we see that 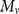 is the reverse mutual inductance for the mode 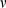. However, from Fig. 3(a), (8), and (19), we see that the forward mutual inductance for the mode 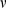 is not 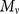, but
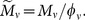
(25)


The ratio (1) follows for 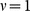. The analogous ratio of forward-to-reverse mutual inductances for other equivalent circuits generated by (13) is
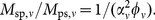
(26)


**Figure 5:**
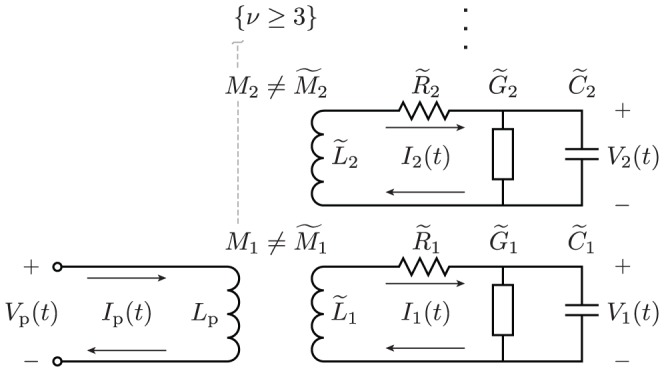
Exact equivalent circuit for the specific example. The coupling with the primary inductor 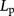 of Fig. 3(b) stitches together the lumped-element models of Fig. 3(a) into this single circuit. The narrowband circuit in Fig. 1(d) is an approximation of this circuit that ignores losses and the contributions of the modes 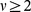, which are nonresonant for frequencies near the fundamental 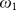.

The exact equivalent circuit for the specific example is sketched in Fig. 5, and is the result of the couplings above stitching together the circuits in Fig. 3. The circuit in Fig. 1(d) is then an approximation that ignores losses (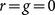) and the contributions of the modes 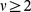. For a spatially uniform current, 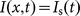 with 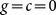, one may use 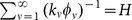 to show that the full circuit in Fig. 5 simplifies to the dc circuit in Fig. 1(b), giving 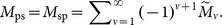



### Standard equivalent circuits for lines

**Figure 6:**
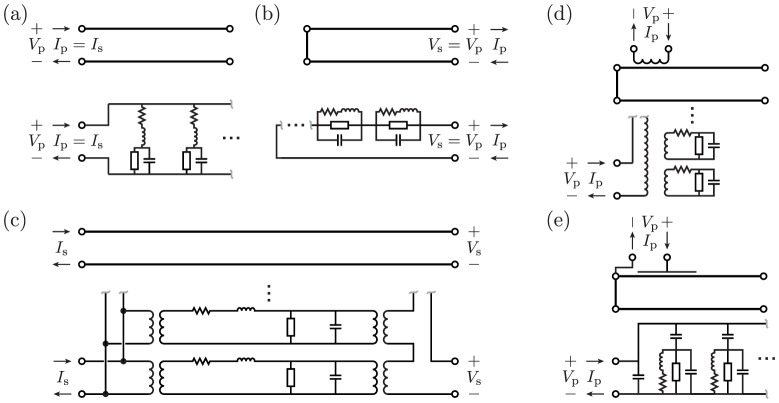
Transmission lines with standard equivalent circuits. These circuits are formed by external coupling stitching together the circuits of Fig. 3, as described in the text. (a) Open-circuit line from direct bottom coupling. (b) Closed-circuit line from direct top coupling. (c) Segment of line from direct top and bottom couplings, such as a coaxial cable with BNC terminals. Note that this circuit is not standard, but follows from the text. (d) Analog of loop-coupled microwave cavity from inductive coupling, or reciprocal version of Fig. 5. (e) Analog of probe-coupled microwave cavity from capacitive coupling.

The approach outlined above differs from those commonly found in textbooks to derive similar circuits. The main difference is that to study Drude's prediction, we did not implicitly assume reciprocity. Nevertheless, one can recover many standard equivalent circuits for lines and their microwave analogs [13]–[15] from the above approach using substitutions (13) with 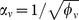. For convenience, these circuits are sketched in Fig. 6 (c.f. Figure 11.12 of Ref. 15). The seemingly unrelated topologies of these various circuits may be graphically understood by noting that they each originate from the circuits of Fig. 3, which stitch together differently depending on coupling with external systems. A direct bottom coupling with 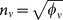, and a direct top coupling with 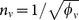 simplify to typical Foster-form circuits for the input impedances of short- and open-ended lines (Fig. 6(a) and (b), respectively). (Half-wave Fourier series are more convenient than (3) here.) Simultaneous top and bottom direct couplings reproduce a segment of line, such as a length of coaxial cable, although this circuit is not standard (Fig. 6(c)). One can show that this circuit reproduces a quarter-wave impedance transformer near resonance. Additionally, inductive coupling with 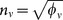 and capacitive coupling with 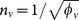 simplify to circuit forms typical for loop- and probe-coupled microwave cavities (Fig. 6(d) and (e), respectively) [13], [28].

## Applications

While the phenomenon described above is likely a rare curiosity, it may be present with resonant single-layer solenoids and Tesla transformers, as first predicted by Drude. However, the conventional modeling of both of these systems has changed since 1904. To describe how this phenomenon may still apply in modern equivalent circuits today, both of these applications are discussed in the next two sections.

Before continuing, it is important to note that the phenomenon behind Drude's prediction is not essential to the modeling of coupling with external systems, or scattering through the line when there is coupling with multiple external systems. For example, the nonreciprocities in Fig. 5 are not required to model the input impedance of the primary inductor in the specific example. Instead, one may use (13) to remove the nonreciprocities in Fig. 5 and produce Fig. 6(e), but at the cost of modeling different voltages and currents than originally intended. Additionally, note that the scope of the approach above is restricted to systems that behave as uniform transmission lines. Other issues with reciprocity may arise in more complex systems such as microwave waveguides [29], [30].

### Single-layer solenoids

To account for stray capacitance, single-layer solenoids and other inductors have been modeled with circuits similar to Fig. 1(d) for over a century [7], [31]–[33]. In the early 20th century, a typical constraint was to set 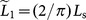 in these circuits, the same as (7) in the specific example [34]. Drude, for example, derived this constraint in an article from 1902 [2], but did not recover it in 1904 [4] because of errors, as shown in Ref. 18. Perhaps this constraint may partly explain why some early texts, such as Hund [32], made a greater allowance for nonreciprocity than is customary today.

Since then, however, the standard constraint has been to use the dc self-inductance, 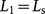 (or 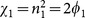), because this conveniently leads to an empirical "self-capacitance'' for a solenoid that is nearly constant over a wide frequency range, after the effects of a capacitive load are included (e.g., following Miller [35]) [36]–[38]. This constraint does not require energy to be modeled correctly or uniquely determine the circuit, except in the low-frequency limit of a spatially uniform current (i.e., infinite load). Thus such circuits may require nonreciprocity as described above. For example, the substitution (13) with 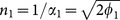 leads to one such circuit that models the current 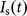 near resonance, for which the ratio (26) of mutual inductances is 1/2. In practice, note that capacitive loads will attenuate or suppress this phenomenon, and again that lines are only approximate models for real solenoids [24].

### Tesla transformers

The conventional equivalent circuit for a Tesla transformer contains a circuit with the same form as Fig. 1(d). Today, this circuit by default uses the dc-inductance constraint described above, despite it not being part of Drude's derivation in 1904 [4]. Importantly, this circuit is nearly always assumed both to be reciprocal and to model the base current 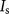 and output voltage 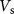 of the secondary solenoid before any spark discharge [8], [9]. Were the current spatially uniform in the solenoid, these three constraints would be compatible. Instead, the current is often nonuniform because typically only a weak external capacitive load is present across the solenoid. The conventional circuit is thus usually overconstrained. Interestingly, this has been observed numerically by enthusiasts who predict nonunique circuit parameters, but did not consider reciprocity [39].

Depending on which of the three constraints are kept, the phenomenon described above may be present. To show what effects this may have, note that the traditional procedure to calculate the maximum possible output voltage follows from conservation of energy and the assumption of a reciprocal mutual inductance, and gives 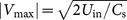 for an energy 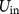 input during operation [40]. Here, 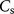 is the sum of the empirical self-capacitance for the secondary solenoid with the capacitance of any loads, such as an output electrode.

This traditional procedure will be inaccurate for weakly loaded or unloaded Tesla transformers, because the standard dc-inductance constraint misrepresents energy in circuits that model 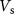. Instead, the ratio of mutual inductances must be included to give the correct result: 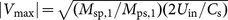. For the unloaded case, using (13) with 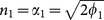 leads to such a circuit that models 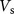 near resonance. Using (26), the ratio 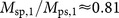 for this circuit, which produces a correction of about 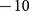
% to the traditional estimate of 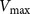. Note that the same correction results if instead the dc-inductance and reciprocity constraints are kept, because of a misrepresentation of output voltage. Using (13) with 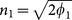 and 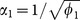 leads to such a circuit, for which 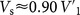. In practice, increasing the capacitive load will quickly reduce the size of this correction, as the current along the secondary solenoid becomes more uniform. For weak loads, this correction may also be obscured by the nonlinear dependence of 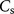 with the capacitive load [31], [33], [35].

## Discussion

As illustrated above, Drude's prediction in 1904 that the mutual inductance should be nonreciprocal for an unloaded Tesla transformer is correct. However, this nonreciprocity assumes that the secondary solenoid acts as a transmission line, and is only present when the current is nonuniform in the solenoid. Even then, it seems that this nonreciprocity will have a relatively small effect, one that may be difficult to measure. Perhaps this is another reason why Drude's prediction is nearly forgotten today.

The phenomenon behind Drude's prediction is a fascinating artifact of modeling distributed transmission lines with lumped equivalent circuits. The resulting nonreciprocity is purely artificial and results only from constraints imposed on equivalent circuits that are incompatible with representing energy correctly. In the specific example, which follows Drude, the incompatible constraint was to model the voltage drop across and current into a resonant inductor—a choice that at first glance may seem straightforward and reasonable. Even today, this constraint is still used in the equivalent circuits of Tesla transformers (e.g., [8]–[10]). Therefore, some care is required to check that the constraints imposed or assumptions made about an equivalent circuit are compatible, otherwise this phenomenon may occur. On the other hand, one may always avoid this phenomenon by constraining a circuit to model energy correctly, as is common today, with the possible cost of breaking other desirable constraints as shown in the specific example.

In summary, distributed systems are not lumped. Modeling transmission lines and analogous systems with lumped equivalent circuits thus creates an opportunity for confusion if the lumped perspective is over emphasized. As Paul Drude predicted in 1904 for Tesla transformers, such systems may require an artificial nonreciprocity to model couplings with other systems when their constraints lead to a misrepresentation of energy, despite all components being reciprocal. This curious, long overlooked prediction is indeed correct, despite its modern appearance.
